# Isolation and identification of antioxidative peptides from crocodile meat hydrolysates using silica gel chromatography

**DOI:** 10.1038/s41598-022-16009-5

**Published:** 2022-08-02

**Authors:** Liu Yang, Yan Xing, Rui Chen, He Ni, Hai-Hang Li

**Affiliations:** Guangdong Provincial Key Lab of Biotechnology for Plant Development, School of Life Sciences, South China Normal University, Guangzhou, 510631 China

**Keywords:** Biochemistry, Biological techniques, Zoology

## Abstract

Crocodiles are cultured in large numbers in Asia and other places in order to protect wild resources and meet the needs of human life. In this study, crocodile (*Crocodylus siamensis*) meat proteins were extracted and hydrolyzed into peptides, their antioxidant peptides were isolated and purified by silica gel chromatography and identified by LC/MS. Crocodile meat proteins were optimally extracted with water and hydrolyzed by papain based on the degree of hydrolysis and antioxidant activity. The hydrolysates were fractionated by ultrafiltration into 3 kDa, 3–30 kDa, and ≥ 30 kDa fractions. The 3 kDa fraction showed most antioxidant activity of the hydrolysates. Its active peptides were separated by silica gel column chromatography and purified by silica gel TLC, based on TLC bio-autographic assays of the activity. Four highly active peptides were identified by LC/MS as SSLTIQFVEGQFVDSYDPTIENTFTK, VPPHIY, VAPEEHPVLLTEAPLNPK, and RNGLPGPIGPAG. The identified peptides were synthesized and showed 50% free radical scavenging activities at 1.0 mg/mL, equal or higher to ascorbic acid at 0.5 mg/mL, in both DPPH and ABTS assays. The results indicated that the 3 kDa hydrolyzed peptides of crocodile meat had high antioxidant activity and the active peptides can be effectively separated and purified by silica gel column chromatography and TLC.

## Introduction

Today, the rapid growth of many non-communicable diseases has aroused the concern of people around the world^1^. Studies have shown that excessive free radicals can cause oxidative stress damage to the human body, and antioxidants can inhibit excessive free radicals^2^. Peptides are one of the main sources of antioxidants, but the mechanism of antioxidant peptides action is not fully clear. There are currently studied surfaces, which may be achieved by removing free radicals, chelating transition metals, and inhibition of lipid peroxidation^3^.

Foodborne antioxidant peptides as an ideal natural alternative are receiving increasing consideration, as chemically synthesized antioxidants showed potential toxicity effects^4^. Among them, animal sources account for a large part. In recent years there have been many related studies, such as goat, sheep milk proteins^5,6^, egg^7,8^ and ham^9^, salmon^10^, duck blood^11^, duck meat hydrolysates^12^, mackerel^13^.

At present, the preparation methods of antioxidant peptides mainly include extraction, fermentation, synthesis, and enzymatic hydrolysis. Among them, considering the cost and repetitive, enzymatic hydrolysis is the most commonly used methods^14^. However, at the same time, we should pay attention to the disadvantages of enzymatic hydrolysis, such as low efficiency and long enzymatic hydrolysis time^15^.

The enzymolysis products of protein is a mixture. At present, according to the molecular weight and net charge of enzymolysis products, the separation and purification technology of peptides mainly include membrane separation, chromatography, and combinations of these technologies^2,16–20^. Identification of protein hydrolysates is usually accomplished by using MS, MS/MS, and bioinformatics techniques. The isolation and purification process of antioxidant peptides have the disadvantages of time consuming and high cost^14^, therefore, more efficient separation and purification methods need to be developed.

Crocodile (*Crocodylus siamensis*) is one of the oldest animals known to live with opportunistic bacteria without overt physiological effects^21^. Crocodiles live in aquatic environments, which abound in pathogenic microorganisms. The animals are often injured in fights or whilst tackling prey, yet appear to show little effects of infection^22^. Therefore, crocodiles are an excellent source of active peptides. In recent years, crocodiles have been farmed on a large scale in China and some countries in Southeast Asia for commercial use, which provides abundant resources for related research. To sum up, we prepare to hydrolyze crocodile meat to obtain antioxidant peptides.

## Materials and methods

### Materials

The crocodile (*Crocodylus siamensis*) meat was purchased from the Huarun supermarket, Guangzhou, China. The meat was brought in an ice box to the laboratory and refrigerated at − 20 °C before use. The silica gel and silica gel G plates (thickness 0.25 mm, 100 × 100 mm or 200 × 100 mm) were purchased from Qingdao Marine Chemicals Co. (Qingdao, China). Protein marker is purchased from Takara Co. (Shanghai, China). Coomassie brilliant blue R-250 and G250, DPPH (diphenyl picryl hydrazinyl radical), ABTS [2,2′-Azinobis-(3-ethylbenzthiazoline-6-sulphonate)], and BCA (bicinchoninic acid) were purchased from Qihuashen Co. (Guangzhou, China). Papain (2.0 × 10^5^ U/g), pepsin (2.3 × 10^5^ U/g), trypsin (1.5 × 10^5^ U/g) and alcalase (3.0 × 10^5^ U/g) were purchased from Qihuashen Co. (Guangzhou, China). Other chemical and biochemical reagents are analytical or biochemical grade and were purchased from local suppliers.

### Preparation of crocodile meat proteins and hydrolysates

#### Degrease

The crocodile (*Crocodylus siamensis*) meat was cut into small pieces, homogenized at room temperature for half an hour and then defatted as described by Jang et al.^23^. The fat in the homogenate of crocodile meat was removed by stirring with isopropanol (1:4, w/v) for 50 min at room temperature. After centrifugation at 700*g* for 30 min, the supernatant was discarded, and the precipitate was lyophilized in a freeze dryer as the defatted crocodile meat.

#### Protein extraction

Five grams of the defatted crocodile meat powder were extracted with 20 ml distilled water and stirred for 10, 20, 40 or 80 min, at room temperature or on boiling water bath, and then centrifuged at 1600*g* for 20 min. The supernatant was taken as protein extracts for enzymatic digestion.

#### Protein hydrolyzation

The crocodile proteins were hydrolyzed according to the previously described procedures^24^ with slight modifications. The protein extracts equivalent to 5 g of crocodile meat was diluted to 100 mL with distilled water and zymolized for 2 h at their corresponding optimal pH and temperature conditions by papain (pH 6.2, 37 °C), pepsin (pH 2.0, 37 °C), trypsin (pH 7.6, 37 °C) and alcalase (pH 8.0, 50 °C). The addition of protease was 2%. After reaction, the hydrolysates were adjusted to pH 7.0 and heated at 97 °C for 10 min to deactivate the enzymes. The hydrolysates were obtained by centrifuged at 1600*g* for 20 min. The supernatant was freeze-dried and stored at − 20 °C.

### Protein analysis

#### Protein contents

The protein contents were determined using the Bradford assay^25^.

#### Degree of hydrolysis (DH)

The degree of hydrolysis of the hydrolysates was determined using the TNBS (trinitro benzene sulfonic acid) method outlined in Maux et al.^26^.

#### SDS-PAGE and Tricine-SDS-PAGE analyses of proteins and peptides

Crocodile meat proteins were analyzed using SDS-PAGE method according to Babini et al.^27^. Stacking and separation gels were 5% and 12%, respectively. All samples with 2% (v/v) 2-mercaptoethanol were heated for 5 min in a boiling water bath. Ten microliter of sample solution was loaded into sample well and electrophoresed at constant voltage of 80 V for stacking gel and 120 V for separating gel. The gel was stained using Coomassie Brilliant Blue G-250.

The hydrolysates were analyzed using Tricine-SDS-PAGE method, according to the method of Li et al.^28^. Stacking and separation gels were 4% and 20%, respectively. All samples with 2% (v/v) 2-mercaptoethanol were heated for 5 min in a boiling water bath. Ten microliter of sample solution was loaded and electrophoresed at constant voltage of 30 V for stacking gel and 100 V for separating gel. The gel was stained using Coomassie Brilliant Blue G-250.

### Determination of the antioxidant activities

The DPPH radical-scavenging activity was determined according to the method of Santos et al.^29^ with some modification. Samples were diluted to 3, 6, 9, 12 and 15 mg/mL with 0.01 mol/L phosphate buffer (pH 7.0). To a 160 μL aliquot of each sample, 640 μL of 0.1 mmol/L DPPH in ethanol (DPPH was dissolved in 95% ethanol) was added. The solution was well mixed and left at ambient temperature for 30 min in the dark. After that, the absorbance of the solution was spectrophotometrically measured at 517 nm. The absorbance value of the sample mixed with DPPH in ethanol was presented as A_sample_. The absorbance value of sample mixed with 95% ethanol solution was presented as A_control_. The absorbance value of DPPH in ethanol alone was presented as A_blank_. The percentage of DPPH scavenging activity was calculated as: Radical scavenging activity (%) = [1 − (A_sample_ − A_control_)/A_blank_] × 100.

The ABTS radical scavenging activity was measured according to the method of Zhang et al.^30^. Briefly, 7 mmol/L ABTS solution was well mixed with 2.45 mmol/L potassium persulfate solution in equal volume, and then the solution was left overnight (12–16 h) in the dark at room temperature. The above solution was diluted with 5 mmol/L phosphate buffer (pH 7.4) to make the absorbance by spectrophotometer at 734 nm be 0.7 ± 0.02. Twenty microliters of sample solution with 3, 6, 9, 12 and 15 mg/mL, was added to 2 mL diluted ABTS reagent, and the mixture was left at ambient temperature for 6 min accurately. Absorbance was then recorded at 734 nm. The blank was prepared in the same way, except that sample was replaced with deionized water. The percentage of ABTS radical scavenging activity was calculated as follows: Radical scavenging activity (%) = (A_blank_ − A_sample_)/A_blank_ × 100.

#### TLC-bioautography

Following the chromatographic separation and evaporation of the mobile phase, the silica gel plates were sprayed equably by chromogenic reagent according to the method of Krüger et al.^31^. Spraying ninhydrin alcohol solution (0.5%, w/v), peptide bands emerged red in 30 min at 105 °C; spraying DPPH alcohol solution (0.1%, w/v), antioxidant bands emerged white over night at room temperature; spraying copper sulfate (1%, w/v), BCA aqueous solution (5%, w/v), protein bands emerged blue over night at room temperature.

### Separation and purification of peptides

#### Molecular weight fractionation

The hydrolysates were fractionated by using ultrafiltration membranes according to Abdelhedi et al.^32^ with molecular weight cut-off (MWCO) membranes of 3 kDa and 30 kDa. The obtained fractions were < 3 kDa, 3–30 kDa, > 30 kDa. The collected fractions were freeze dried, stored at − 20 °C.

#### Separation of active peptides by silica gel column chromatography

Active fraction of MW < 3 kDa was dissolved in 1 ml distilled water and loaded onto a silica column (Φ2.7 × 40 cm). The column was eluted sequentially with solvents of 1,2-dichloroethane/methanol of 7/3, 6/4, 5/5, 4/6, 3/7, 2/8, 1/9, 0/10, and methanol/water of 9/1, 50/50 and 0/10), each for one bed volume, at a flow rate of 2.0 ml/min. The fractions were concentrated and stored at − 20 °C until used.

#### Analysis and purification of antioxidant peptide by TLC

The samples or active fractions from silica column chromatography were analyzed or purified by TLC. Silica gel TLC plates were developed/washed with methanol and then activated at 105 °C for 30 min, before used for sample separation. After loaded the samples, the TLC plates were developed with solvents of 1,2-dichloroethane: methanol:ammonia 1:9:0.2 (V/V).

### Identification and synthesis of the purified peptides

Purified peptides were identified by HPLC–MS in the Wininnovate Company (Shenzhen, China). Identified active peptides were synthesized by NJ peptide Company (Nanjing, China) for activity assay.

### Statistic analysis of the data

All quantitative analyses were repeated at least three times. The averages plus standard errors were presented.

## Results and discussion

### Preparation crocodile meat protein and its hydrolyzed peptides

**Figure 1:**
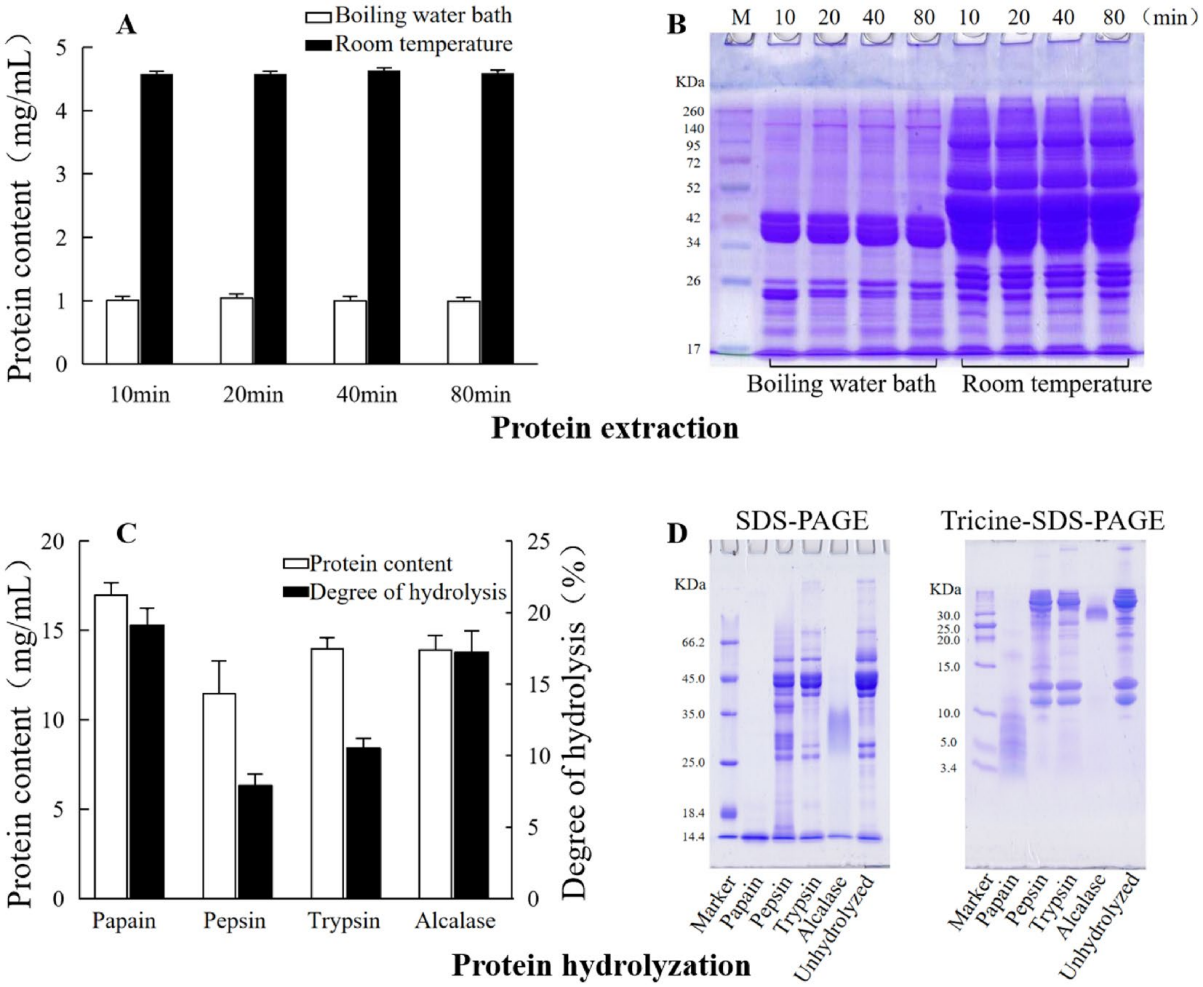
Extraction and hydrolysis of crocodile meat proteins. (**A**) Soluble protein contents extracted with water at room temperature or on boiling water bath; (**B**) SDS-PAGE analysis of soluble proteins extracted at temperature or on boiling water bath; (**C**) Protein contents and hydrolysis degree of crocodile meat hydrolyzed by different enzymes; (**D**) SDS-PAGE and tricine-SDS-PAGE analyses of the hydrolysates by different enzymes.

Crocodile meat was homogenized and its soluble protein was extracted with water either at room temperature or on boiling water bath. As shown in Fig. 1A, the extracted protein amount was much higher at room temperature than on boiling water bath (Fig. 1A). SDS-PAGE analyses show that much more bands of protein were extracted at room temperature (Fig. 1B). There were no significant differences for extraction times from 10 to 80 min (Fig. 1A,B). Therefore, the optimal protein extraction conditions were determined to be extract with water at room temperature for 10 min.

Four proteases, papain, pepsin, trypsin and alcalase, were tested for their efficiency to hydrolyze the crocodile meat proteins. Degree of hydrolysis (DH) is an important indicator in protein hydrolysis which relates to the size, the amino acid composition and the biological activity of the hydrolyzed peptides^33,34^. DH, together with SDS-PAGE analysis was used to evaluate the hydrolyzed proteins by the four proteases. As shown in Fig. 1C, DHs were lower in the hydrolysates by trypsin (DH 10.2%) and pepsin (DH 8.2%) than those by papain (DH 19.2%) and catalase (DH 17.1%). The hydrolysates by papain had the highest DH. It was reported that antioxidant activity of porcine blood plasma protein hydrolysates increased with increasing DH^33^.

The peptide size distribution in the hydrolysates by four proteases were analyzed by SDS-PAGE and tricine-SDS-PAGE. The results shown that the proteins were not well digested by pepsin and trypsin. The proteins were better but not completely digested by alcalase, and most of its peptides are between 25–35 kDa. Papain digested most of the proteins into small molecular weight peptides, and most of its peptides were less than 10 kDa (Fig. 1D). The results indicated that papain is the best enzyme to digest crocodile meat proteins and was used to make small molecular peptides.

### Separation of the hydrolysates by ultrafiltration

**Figure 2:**
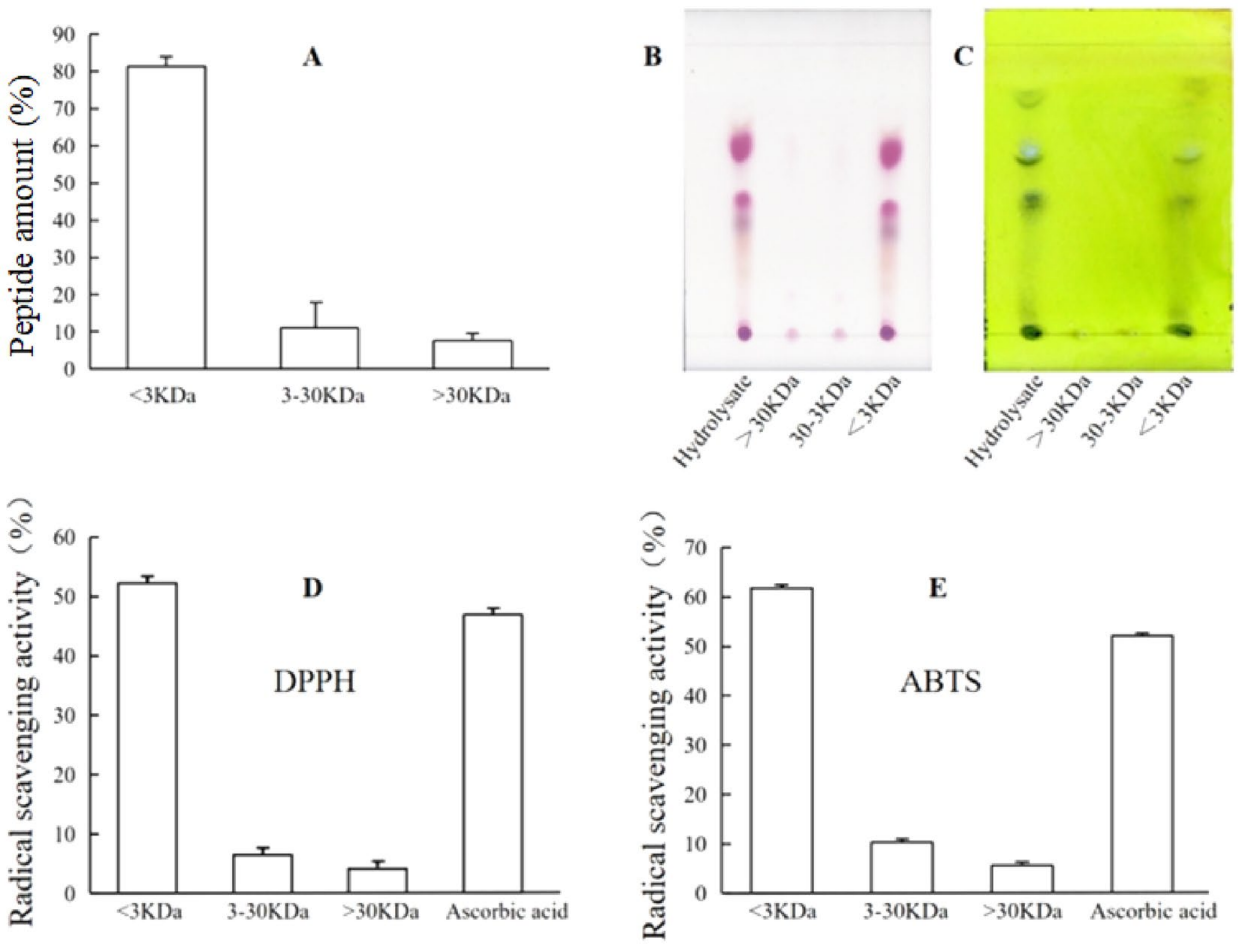
Peptide analysis of the three fractions of crocodile meat hydrolysates after ultrafiltration and their antioxidant activity. (**A**) Peptide amounts in the three fractions after ultrafiltration; (**B**,**C**) TLC analyses of the three fractions of peptides stained by ninhydrin alcohol solution(0.5%, w/v) or by BCA solution(5%, w/v in 1% copper sulfate; (**D**,**E**) antioxidant activities of the peptides against DPPH and ABTS.

The protein hydrolysates of crocodile meat digested by papain were separated into three fractions by ultrafiltration based on their molecular weights (MW). Three fractions with MW less than 3 kDa (3 kDa fraction), between 3 and 30 kDa, and more than 30 kDa were obtained. As shown in Fig. 2A, the 3 kDa fraction accounted for 80% peptide amount of the hydrolysates, which is coincide with its high DH. The three peptide fractions were also analyzed by TLC, stained with ninhydrin for amino acid/peptide (Fig. 2B), or stained by BCA for protein/peptide (Fig. 2C). TLC analysis (Fig. 2B,C) showed similar results as the quantitative analysis of peptides (Fig. 2A).

The antioxidant activities of three peptide fractions were detected quantitatively by the DPPH and ABTS methods (Fig. 2D,E). The results showed that most the antioxidant activities were found in the 3 kDa fraction and the radical scavenging activity was 52% in DPPH assays and 62% in ABTS assays. The other two fractions showed very low antioxidant with the radical scavenging activity less than 10% in both DPPH or ABTS assays (Fig. 2D,E). Mejria et al.^35^ and Yang et al.^36^ determined that fractions with smaller molecular weights (< 3 kDa) presented higher hydroxyl radical scavenging activity than those with higher molecular weights. Therefore, only the highly active 3 kDa fraction was selected for further experiments.

### Separation and purification of the active peptides by silica gel chromatography

The silica gel column chromatography (SGCC) is a common and excellent method for adsorptive separation of low polar small molecular compounds. Hoverer, it was considered not good for separation of high polar and water-soluble compounds. The SGCC was tested to isolate active peptides in the 3 kDa fraction of crocodile meat peptides.

**Figure 3:**
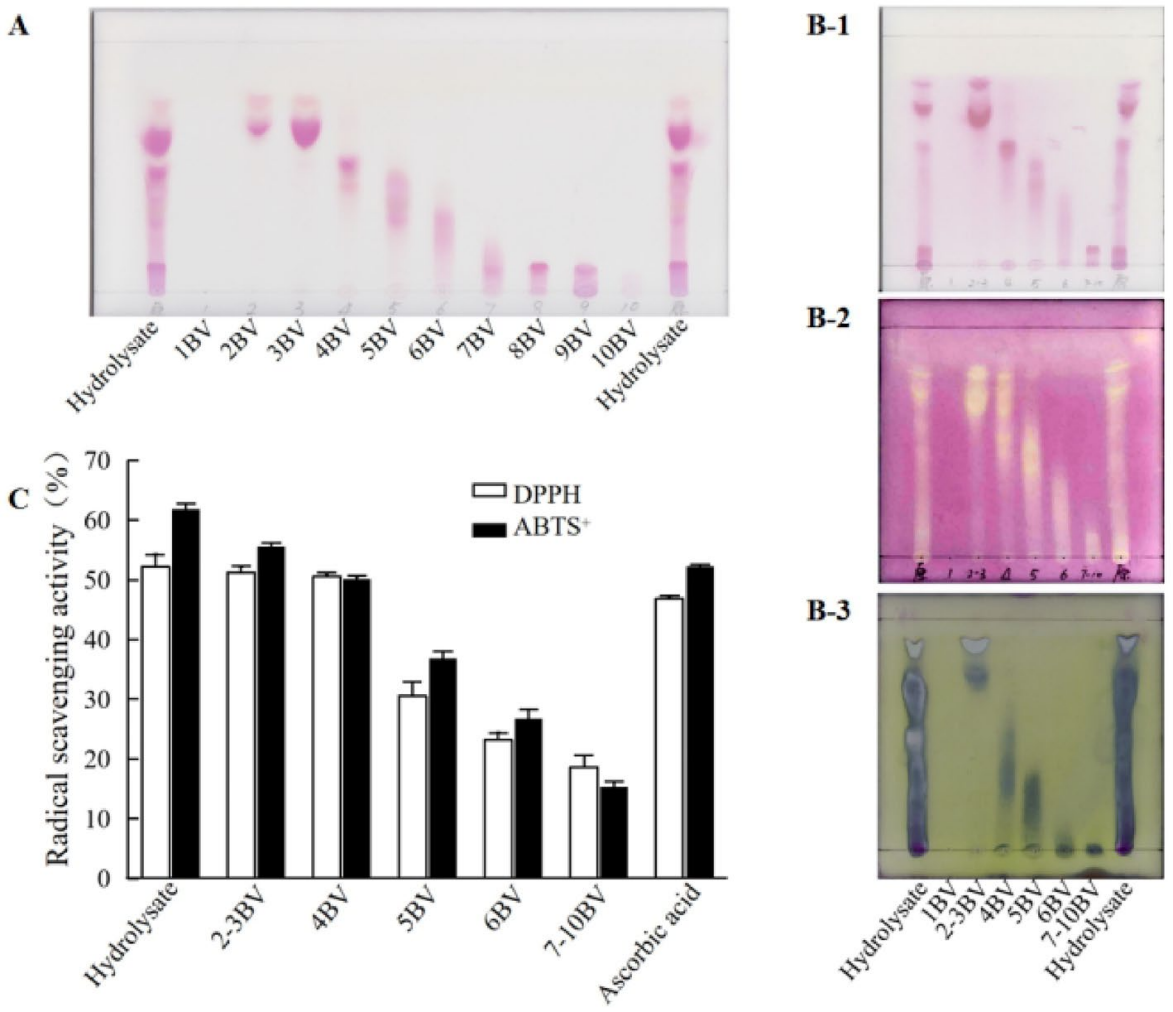
Analysis of the peptide fractions separated by silica gel column chromatography. (**A**) TLC analysis of different fractions of peptides eluted from the column; (**B**) TLC-bioautographic analysis of different eluents of peptides. The TLC plates were stained by ninhydrin (**B-1**), by DPPH (**B-2**), or by BCA solution (**B-3**). (**C**) Quantitative analysis of antioxidant activity of different peptide fractions from the column chromatography, using ascorbic acid (0.5 mg/mL) and the hydrolysates (1.0 mg/mL) as controls.

The 3 kDa fraction of peptides were dissolved in a small volume of water and loaded on the silica gel column and eluted sequentially with increased polar of solvents as described in the materials and method. The eluents were collected in fractions per BV. All eluent fractions were analyzed on TLC and their antioxidant activities were determined (Fig. 3). As shown in Fig. 3A, the peptides were well separated into 10 fractions by the silica gel column chromatography. The 10 fractions were combined into six fractions based on the TLC analysis. The combined fractions were further analyzed on TLC, with ninhydrin staining (Fig. 3B-1) and BCA staining (Fig. 3B-2), and their antioxidant activities were analyzed by TLC-bioautography (Fig. 3B-3) and by quantitative DPPH and ABTS methods (Fig. 3C). Ninhydrin staining shows amino acids and peptides (Fig. 3B-1) while BCA staining shows only peptides (Fig. 3B-2). Comparing the bands in Fig. 3B-1, B-2 with the TLC-bioautographic assay in Fig. 3B-3, the main antioxidant active bands are in the fractions 2–4 BV and most of them are peptides, rather than amino acids. Similar results were obtained in the DPPA and ABTS assays that the 2-3BV and the 4 BV are highly active and other fractions are less active. The 2–4 BV were combined together for further purification of active peptides by TLC.

**Figure 4:**
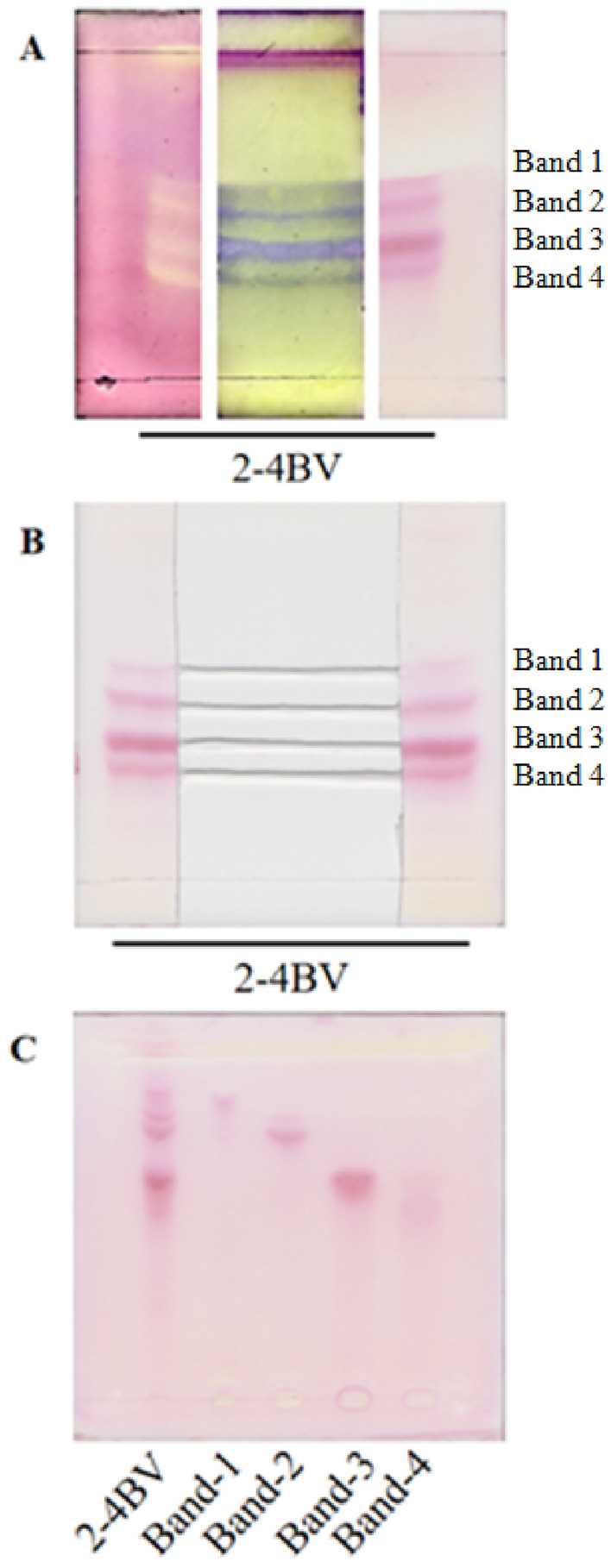
Purification of the active peptides by silica gel TLC in the 2–4 BV combinations from silica gel column chromatography based on TLC-bioautography. (**A**) TLC analyses of the active peptides. After developed, the TLC plate were cut into three parts and stained by (from left to right) DPPH, BCA solution and ninhydrin solution; (**B**) Separation of active peptides by scraping the active bands from the TLC plate. (**C**) TLC purified peptides.

The 2–4 BV from the SGCC was further separated on silica gel TLC plates. The sample was loaded on the TLC plate in line. After developed with 1,2-dichloroethane/methanol/ammonia = 3/7/0.2, the plate was cut into three pieces and stained with DPPA (left), BCA (middle) or ninhydrin (right) as shown in Fig. 4A. Four clear peptide bands can be recognized. On the separating TLC, two sides of the plate were cut off and stained with ninhydrin, and the silica gel in middle part was used for scraping off the peptide samples (Fig. 4B). The peptides in the silica gels were extracted with water and analyzed on TLC. The four peptide bands were well separated (Fig. 4C), which indicated that peptides can be effectively separated by the silica gel TLC.

### Identification of active peptides by LC–MS and their activity analysis

The peptides isolated from the TLC plate were analyzed and identified by LC–MS. The detected peptides were screened, deleting the peptides with low amount and from microorganisms (microbial contaminated) or enzyme fragments. From each active band (Fig. 4B), one peptide with high amount and originated from muscle structural proteins based on Uniprot Knowledgebase (UniProtKB) was selected as potential active peptide. The four potential active peptides were identified as SSLTIQFVEGQFVDSYDPTIENTFTK (UniProtKB Accession number C0HM48) from band 1, VPPHIY (C0HM49) from band 2, VAPEEHPVLLTEAPLNPK (C0HM50) from band 3 and RNGLPGPIGPAG (C0HM51) from band 4.

The four identified peptides were synthesized for the study of their antioxidant activities. The synthesized peptides were confirmed for their amino acid sequences by LC–MS. Their antioxidant activities were quantitatively detected using ascorbic acid (0.5 mg/mL) and the crude peptide hydrolysates (1.0 mg/mL) as positive controls.

**Figure 5:**
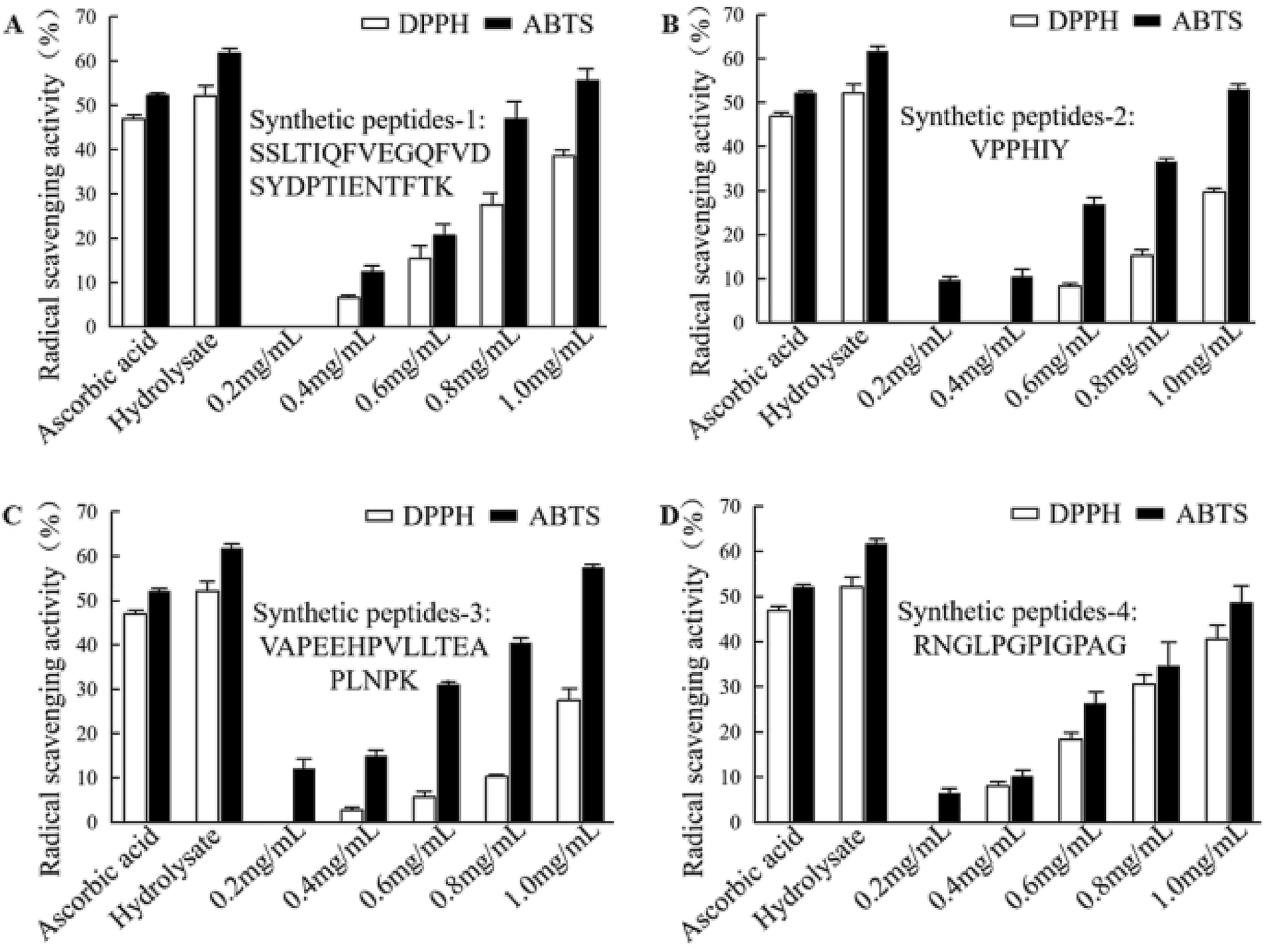
Antioxidant activities of the identified peptides using synthetic peptides. (**A**) SSLTIQFVEGQFVDSYDPTIENTFTK; (**B**) VPPHIY; (**C**) VAPEEHPVLLTEAPLNPK; and (**D**) RNGLPGPIGPAG. Ascorbic acid at 0.5 mg/mL and the hydrolysates at 1.0 mg/mL were used as controls.

All the four peptides showed a concentration dependent antioxidant activity on the scavenging both DPPH and ABTS radicals. They had scavenging activities as low as 0.2 or 0.4 mg/mL, the activities increased with the increase of peptide concentrations. When the peptides concentration at 1.0 mg/mL, all the four peptides showed the same or higher activities as that of 0.5 mg/mL ascorbic acid. The results also showed that the radical scavenging activities of the four peptides were higher to DPPH than to ABTS radical (Fig. 5).

There are many reports on the isolation and identification peptides sequences from animal tissue. Bashir et al. isolated antioxidant peptides from mackerel which ranged from 547.29 to 1049 Da^13^. Choksawangkarn et al. isolated two antioxidant peptides from fish sauce by-product, which were identified as PQLLLLLL and LLLLLLL^17^. Mejria et al. identified antioxidant peptides by RP-HPLC-Q-TOF-MS in dry fermented camel sausages^35^. Yang et al. found seven novel antioxidant peptides from duck plasma hydrolysate: LDGP, TGVGTK, EVGK, RCLQ, LHDVK, KLGA, and AGGVPAG^36^. Shazly et al. identified the antioxidant peptides by LC MS/MS in buffalo casein hydrolysates: RELEE, MEDNKQ and TVA, EQL^37^. The LC/MS method for identification of peptides have been demonstrated to be an effective method.

## Conclusion

In this work, antioxidant peptides were prepared and analyzed, and four antioxidative peptides were purified and identified from crocodile meat hydrolysates. The results indicated that the proteins of crocodile meat were extracted with water and hydrolyzed by papain, which can make the most active peptides. The hydrolysates were ultrafiltrated into high (≥ 30 kDa), medium (3–30 kDa) and small molecular weight (≤ 3 kDa) fractions, and the 3 kDa fraction had most antioxidant activity of the hydrolysates. After separation by silica gel column chromatography and purification by silica gel TLC from the 3 kDa fraction, four highly active peptides were identified by HPLC–MS as SSLTIQFVEGQFVDSYDPTIENTFTK, VPPHIY, VAPEEHPVLLTEAPLNPK and RNGLPGPIGPAG. All the four peptides showed content dependent antioxidant activity, and can scavenge 50% or more DPPH and ABTS radicals at 1.0 mg/mL, which is equal or higher than that of 0.5 mg/mL ascorbic acid. The results indicated that the hydrolyzed peptides of crocodile meat by papain had high antioxidant activity. The peptides can be effectively separated by common silica gel column chromatography and purified by silica gel TLC, following the TLC-bioautographic assay of antioxidant activity.

## Data Availability

The datasets used and/or analysed during the current study available from the corresponding author on reasonable request.
